# Supplementary material for: Ligand Design Using Unique Conformations to Preferentially Dock a Specific Site on Collagen-Bound MMP1
Source: Biology (Basel). 2026 Jul 16;15(14):1169. doi: 10.3390/biology15141169 (PMC13403677; doi:10.3390/biology15141169)
Supplement: Supplementary file 1 [file biology-15-01169-s001.zip › biology-4351117-supplementary.pdf]

Supplementary Information

Ligand design using unique conformations to preferentially dock a specific site on collagen-bound MMP1

Anthony Nash, Chase Harms, and Susanta K. Sarkar*

[1]School of Molecular Sciences, Arizona State University, Tempe, AZ.

**Corresponding author: susanta.sarkar@asu.edu*

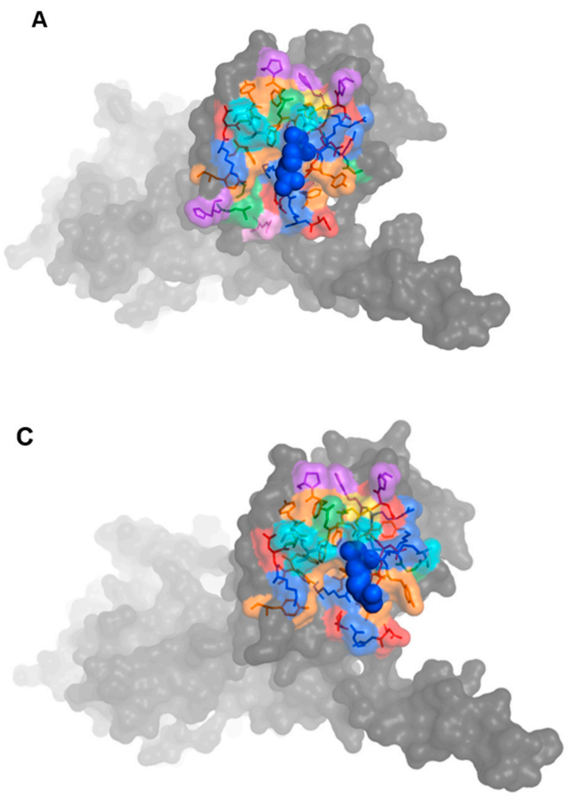

Figure S1. Dynamic collagen-specific allosteric fingerprints on MMP1. Representative conformations of fingerprint clusters corresponding to (**A**) #370, (**B**) #375, and (**C**) #405. Collagen-bound MMP1 is shown as a gray surface, with R405 highlighted as blue spheres. Residues within the local fingerprint environment are shown as sticks and colored according to side-chain chemistry: acidic (red), basic (blue), aliphatic (green), sulfur-containing (yellow), heterocyclic (purple), hydroxyl-containing (cyan), aromatic (orange), and amide (pink).

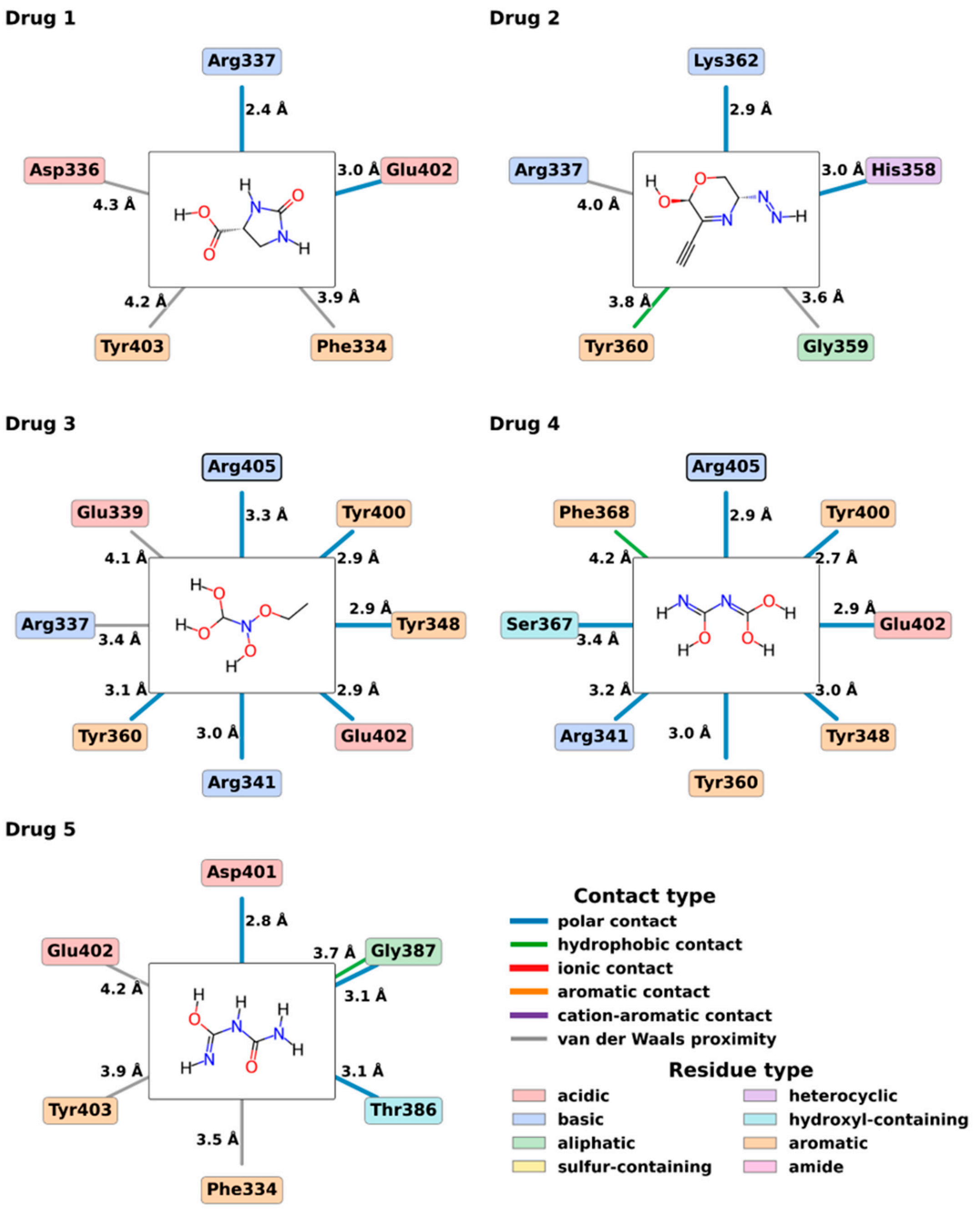

Figure S2. Chemical structures and docked ligand-receptor interaction maps. Chemical structures of the five top-ranked compounds and their two-dimensional docked ligand-receptor interaction maps are shown. Each panel shows a docked compound together with neighboring receptor residues, labeled and colored according to side-chain chemistry. Interaction distances are shown in Å. Contact lines indicate the predicted interaction type, including polar, hydrophobic, ionic, aromatic, cation–aromatic, and van der Waals interactions. Residue colors denote the classes of acidic, basic, aliphatic, sulfur-containing, heterocyclic, hydroxyl-containing, aromatic, and amide side chains.
